# Usability evaluation of an integrated electronic medication management system implemented in an oncology setting using the unified theory of the acceptance and use of technology

**DOI:** 10.1186/s12911-020-01348-y

**Published:** 2021-01-06

**Authors:** Racha Dabliz, Simon K. Poon, Angus Ritchie, Rosemary Burke, Jonathan Penm

**Affiliations:** 1 Faculty of Medicine and Health, School of Pharmacy, The University of Sydney, Sydney, NSW Australia; 2 School of Computer Science, University of Sydney, Sydney, NSW Australia; 3 Concord Clinical School, University of Sydney, Sydney, NSW Australia; 4 Health Informatics Unit, Sydney Local Health District, Camperdown, NSW Australia; 5 Pharmacy Services, Sydney Local Health District, Camperdown, NSW Australia; 6 Department of Pharmacy, Prince of Wales Hospital, Randwick, Australia

**Keywords:** Electronic medication management system, Medication safety, Hospital, Oncology, Usability, Theory, Unified theory and use of technology

## Abstract

**Background:**

Medication management processes in an Oncology setting are complex and difficult to examine in isolation from interrelated processes and contextual factors. This qualitative study aims to evaluate the usability of an Electronic Medication Management System (EMMS) implemented in a specialised oncology unit using the Unified Theory of Acceptance and Use of Technology (UTAUT) framework.

**Methods:**

The study was conducted in a 12-bed outpatient Oncology unit of a major teaching hospital 6 months following implementation of a commercial EMMS. In-depth semi-structured interviews were conducted with doctors, nurses and pharmacists using the system to assess usability. The UTAUT framework was used to analyse the results, which facilitated evaluation of interrelated aspects and provided a structured summary of user experience and usability factors.

**Results:**

Direct cross-comparison between user groups illustrated that doctors and pharmacists were generally satisfied with the facilitating conditions (hardware and training), but had divergent perceptions of performance (automation, standardised protocols and communication and documented) and effort (mental and temporal demand) expectancy. In counterpoint, nurses were generally satisfied across all constructs. Prior experience using an alternative EMMS influenced performance and effort expectancy and was related to early dissatisfaction with the EMMS. Furthermore, whilst not originally designed for the healthcare setting, the flexibility of the UTAUT allowed for translation to the hospital environment.

**Conclusion:**

Nurses demonstrated overall satisfaction with the EMMS, whilst doctors and pharmacists perceived usability problems, particularly related to restricted automaticity and system complexity, which hindered perceived EMMS success. The study demonstrates the feasibility and utility of the UTAUT framework to evaluate usability of an EMMS for multiple user groups in the Oncology setting.

## Background

Electronic Medication Management Systems (EMMS) that support cancer care must consider key areas of practice that differentiate oncology from other medical specialties. Result flowsheets, the need for multidisciplinary workflow documentation, integration of laboratory and imaging reporting, and dealing with time-dependent, patient-specific chemotherapy dosing as well as supportive medications are some of these unique demands [1, 2]. Particularly demanding is the ordering of complex chemotherapy regimens, documentation, and workflow management functionalities such as multiple authorisations and checks of cytotoxic chemotherapy orders by an oncologist and verified by pharmacists and nurses [1, 2].

Studies of EMMS implementations in the oncology setting have found that critical success factors include; the design and usability of the EMMS [3], standardization of chemotherapy protocols [3–6], seamless integration with other health information systems and user workflows [2–5, 7–11], effective training and support [4, 12], support from leadership [4, 12, 13], collaborative project management [13], and effective ongoing maintenance and support [9, 13, 14].

Usability can be defined as the extent to which a product can be used by specific users to achieve specific goals, with effectiveness, efficiency, and satisfaction in a specific context of use [15]. The usability of a technology is determined not only by its user–computer interactions, but also by the degree to which it can be successfully integrated to perform tasks in the intended work environment [15]. Previous studies comparing the user satisfaction of doctors and nurses found different degrees of user acceptance [16–19], however most research on usability has been limited to one type of user through quantitative research.. Usability is evaluated through the interaction of user, system, and task in a specified setting. Several theories that measure individual and organizational acceptance and success have been designed and validated [20].

The Unified Theory of Acceptance and Use of Technology (UTAUT) is widely used to evaluate usability in the healthcare system [20]**.** However, no study has applied the UTAUT framework to explore usability in the Oncology EMMS setting. Furthermore, previous studies exploring Oncology EMMS have predominately focused on usability from a single user perspective in isolation, such as prescribers [21], EMMS providers [22], user requirements before implementation [23] or its impact on medication safety [2, 24].

In light of the complexity of the medication process and the difficultly of examining it in isolation from other interrelated processes and contextual factors, this study aims to evaluate the usability of an EMMS in the Oncology setting from the perspective of nurses, doctors and pharmacists using the UTAUT model.

## Method

### Sample and setting

This study was undertaken in a 12-bed outpatient Oncology unit in a major teaching hospital in Sydney, Australia. The outpatient oncology unit is attached to a 750-bed teaching hospital treating a range of adult solid organ tumours and haematology malignancies. A commercial EMMS (Cerner Millennium) was implemented in the Oncology unit in June-2018. The EMMS is used for chemotherapy and antineoplastic prescribing and administration. The EMMS is part of a single, integrated Electronic Medical Record (EMR) which contains most other aspects of patient care, including pathology, imaging, tests orders and electronic clinical documentation.

**Fig. 1:**
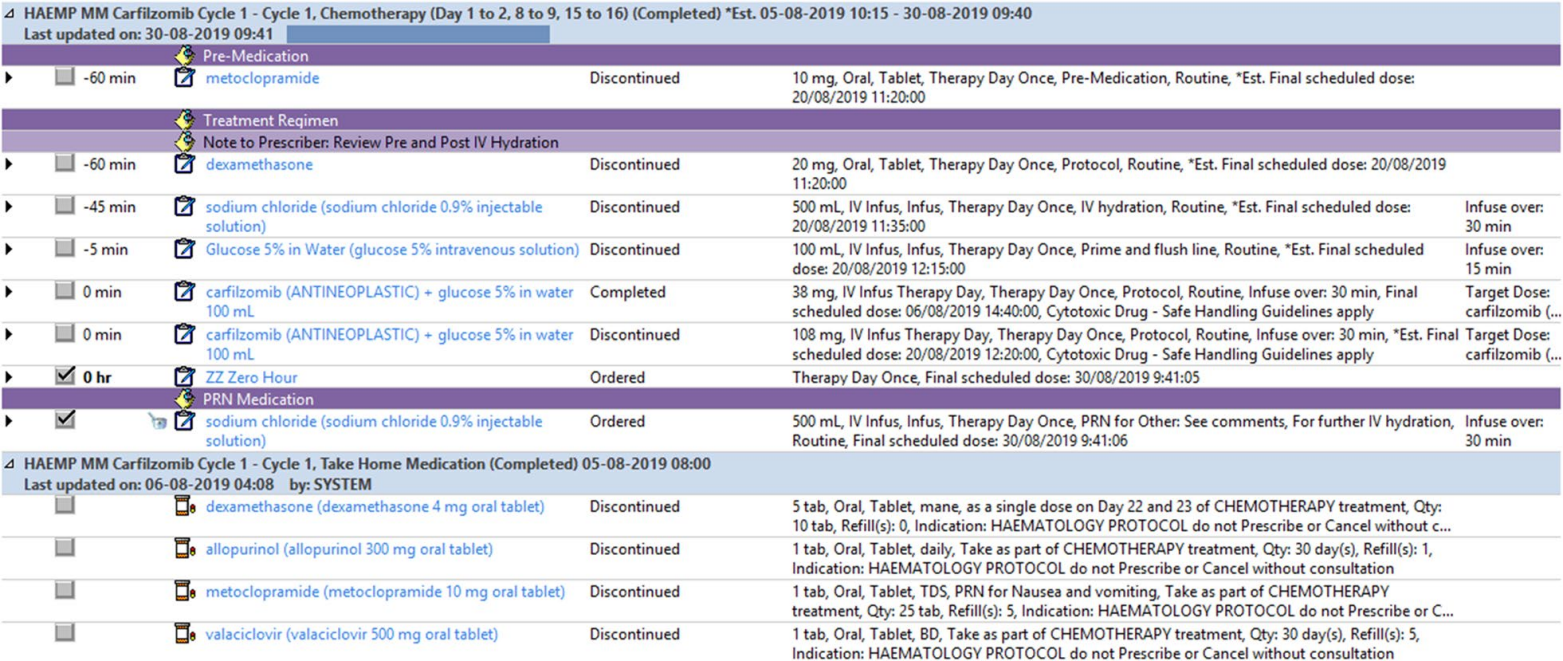
Sample Electronic Medication Management System Oncology specific order sets

The EMMS introduced oncology-specific order sets to ensure that potentially related orders are available in one place for the convenience of the prescriber (Fig. 1). The order sets are designed to comply with best-practice clinical guidelines that are widely used in Australia [25]. Throughout the evaluation, approximately 70% of cancer treatment regimens were managed electronically, whilst the remaining 30% were paper based.

The EMMS allows prescribing, pharmacy verification, and documentation of drug administration and medication reconciliation. All orders are subjected to series of system checks including drug allergies and interactions. Following prescribing, orders appear on a summary view of medications prescribed, as well an administration screen called the Medication Administration Record (MAR). Once orders have been prescribed, the medication protocol auto-populates as a sequence on its calculated due date. Once verified by a pharmacist, it requires activation by the nurse prior to administration.

Participant recruitment began in December 2018, after 6 months had elapsed following implementation, and concluded in May 2019. Delaying evaluation by 6 months after EMMS implementation was to minimise the potential effect of initial user resistance and to allow adoption of new processes.

### Interview guide

Semi-structured interviews were conducted, guided by the constructs of the UTUAT that consisted of 16 question items (Additional file 1), allowing participants to express their attitudes towards the constructs. UTAUT integrates eight theoretical perspectives on technology adoption and provides a comprehensive view of the factors related to users’ adoption behaviour [26]. The main UTAUT constructs are [27]:

- Performance expectancy (PE): “The degree to which an individual believes that using the system will help him or her attain gains in job performance”.
- Effort expectancy (EE): “The degree of ease associated with the use of the system”.
- Social influence (SI): “The degree to which an individual perceives that important others believe he or she should use the new system’.
- Facilitating conditions (FC): “The degree to which an individual believes that an organizational and technical infrastructure exists to support use of the system’.
- Behavioural intention (BI): “The willingness of respondents to use the system.”

The model was chosen as it examines the presence of each construct in a “real world” environment, allowing assessment of an individual’s intention to use a specific system, thus allowing for the identification of the key influences on acceptance in any given context [26].

### Participants and recruitment

Purposive, maximum-diversity sampling was used to recruit nurses, doctors and pharmacists that care for oncology patients. They were expected to have worked in the oncology unit for at least 3-months prior to and during the implementation of the EMMS. For nursing staff, recruitment material was distributed by the ward’s allocated education nurse. Doctors and pharmacists were recruited via email from their heads of department. Recruitment ceased once data saturation for each of the user groups had been achieved. The same researcher (a pharmacist research student) interviewed all user groups. The researcher had not worked closely with interviewees previously or in the Oncology unit. The interviewees were provided with an explanation of the purpose of the study. It was also explained that despite the interviewer also being an employee of the hospital, their role in this study was purely an investigator [28]. The interviewee’s role in the study was also described to them as well as the opportunity to withdraw at any stage of the study [28].

### Data analysis

Inductive and deductive methods [29] were used in the data analyses. Interviews were transcribed verbatim and a process of inductive and iterative transcript analysis methods [30] were applied to identify new or emerging themes. Data collection and inductive thematic analysis were iterative, allowing themes in the data to be explored in depth and contradictory data to be investigated. Inductively identified codes were independently developed by two of the researchers and the differences were resolved through discussion between the coders, assisted using NVivo 11 (QSR International Pty Ltd., released 2015, Version 1.0.1.1). Codes were grouped into overarching themes by the research team and then deductively mapped to the constructs under the UTAUT [31]. The UTAUT framework helped to guide the research; facilitate comparison of findings between user groups and relate these findings to the existing literature. In addition, an ongoing review of the literature on the topic of EMMS implementation enabled us to validate, compare, and extend our findings [32].

## Results

Twenty-seven participants took part in the interviews, representing approximately 79% of all eligible staff. The mean length of interviews was 45 min. The demographic characteristics of the participants are presented in Table 1. Most of the participants (*n* = 23, 85%) were female and included doctors (*n* = 10), pharmacists (*n* = 7) and nurses (*n* = 10). Interviews generated extensive data relating to usability and acceptability of the EMMS. The themes in these categories were mapped to the UTAUT framework (Table 2). The perception of usability related to the various elements of the UTAUT have been described in detail below.

**Table 1:** Demographic of interview respondent – EMMS = Electronic Medication Management System

Measure/user group	Doctor	Pharmacist	Nurse
Frequency	10	7	10
Gender (F/M)	2/ 8	5 / 2	10/0
Age			
24–29	0	2	1
30–34	4	2	0
35–39	1	0	2
40–49	3	1	4
50–59	1	0	3
60+	1	0	0
Years of experience in healthcare			
< 10	5	5	5
10 to 19	3	1	1
20–29	0	1	3
30 or >	2	0	1
Months of experience using the EMMS			
1 to 3 months	1	1	0
4 to 6 months	4	3	0
> than 6	5	3	10

**Table 2:** Interview themes mapped to the Unified Theory of Acceptance and Use of Technology (UTAUT) framework. EMMS = Electronic Medication Management System

	Themes		
	EMMS factors	Organizational factors	Individual factors
Categories under the themes mapped to the UTAUT			
Performance expectancy	Automation and medication safety Standardizing Protocols Communication and documentation		Expectations based on experience
Effort expectancy	Mental Demand Temporal Demand		Expectations based on experience
Social influence		Hospital’s Social Structure	
Facilitating condition		Training Hardware	
Behavioral intention			Benefits Sustainability

### EMMS factors

EMMS factors impacted on the perception of Performance and Effort Expectancy across all user-groups. System factors such as introduced automation, standardized protocols and communication and the downstream impact on mental and temporal demand, had individualised impacts on perceived system usability.

#### Performance expectancy

Overall, all user groups felt that improved automation and standardization introduced by the EMMS led to the improvement of their overall performance. However, doctors and pharmacists expected a greater impact on their performance. They were disappointed by the limited degree of automation, influenced by pre-conceived ideas as well as experience with other systems.

##### Automation and medication safety

Reduced ‘mental energy’ introduced by the EMMS was described by a senior doctor involved in transcribing medication charts. Doctor 1 (Table 3) expressed satisfaction with the automated calculation of Body Surface Area and Area Under the Curve. However, doctors and pharmacists believed the safeguards within the EMMS aren’t adequate to prevent errors or inappropriate prescribing, as described by pharmacist 4 (Table 3).

**Table 3:** User perceptions of elements of performance expectancy extracted from nurses, doctors and pharmacists’ interviews

	Elements of performance expectancy and illustrative quote		
	Automation and medication safety	Standardising protocols and dosing	Communication and documentation
Doctors	*Doctor 1: ‘There’s a certain element of automation of AUCs and BSAs that are done that are handy and dose rounding, it probably has taken some of that load off’.*	Doctor 3: *‘The protocols written in there are set in there … and are established protocols’ … ‘dose variance and looking for significant patterns of care and how you can track that it’s appropriate across a whole department, a whole service I think is definitely valuable’*	Doctor 2: *‘We needed a refresh of the way we communicated, and I think the EMMS provided a trigger point to do that’.*
Pharmacists	Pharmacist 4: *‘A person started chemotherapy on the ward today and now their day 22 and 23 dexamethasone has ended up for tomorrow … that should not happen, I know like it’s probably a bit of user error and computer error, but stuff like that should be prevented by the system’.*		Pharmacist 1: *‘It (the system) always says, what the original or protocol dose would be, so you’d know that it’s different so that is good’.* *Pharmacist 2:* *‘They’re (the doctors) not properly using them, like there’s a lot of information on those templates but it’s just too hard to use them so they put all this info on the one section, so you’ve got this whole page thing but they’re only using one block’*
Nurses		Nurse 3: *‘Less misinterpreting a medication, I feel it’s safer, and it’s just clear, and it’s all there for you. You can see what’s been given and what hasn’t.*’ Nurse 5: *‘The provision of the protocols that are listed, they’re all in sequence so it’s quite easy and allows me to better prepared prior to a patient’s arrival’.*	Nurse 2: *‘We can see the timings like if it’s given by someone, suppose I was looking after the patient and someone comes while I’m on break, so they can easily see which (medication) I’ve already given and what time’.*

##### Standardizing protocols and dosing

Both nurses and doctors appreciated the impact of standardized protocols on their performance. The standardized layout of medication charts was perceived to improve medication safety and nurse’s performance. As they described that there was less chance of chart misinterpretations (nurse 3, Table 3) and a user-friendly layout that supports medication administration (nurse 5, Table 3). Similarly, doctors appreciated that ‘the protocols written in there are set in there … and are established protocols’ (doctor 3, Table 3). The benefits introduced by an automated dose variance report allowed management to oversee unusual prescribing, as described by doctor 3 (Table 3). Collectively, standardization and the ability to track dose variance were perceived as beneficial in improving overall performance.

##### Communication & documentation

Doctors and pharmacists felt that communication and documentation improved to a degree. Pharmacists appreciated the clarity of communication regarding dose reductions as described by pharmacist 1 (Table 3). The system also highlighted original gaps in their workflows that previously existed, indicated by doctor 2 (Table 3). However, there were concerns about the format of the treatment plan documents. The templates were not being used optimally, as described by pharmacist 2 (Table 3). From the nursing perceptive, it greatly improved communication between the multidisciplinary teams and amongst each other, as identified by nurse 2 (Table 3).

#### Effort expectancy

Effort levels for the various user groups drew on various dimensions of effort such as mental demand and temporal demand. Users described varying impacts of the EMMS on these elements of effort and are summarised in Table 4. Doctors expressed satisfaction with the integrated order sets and the reduced time spent tracking previously prescribed regimens, as explained by doctors 3 and 4 (Table 4). However, a lack of system flexibility and complexity had a negative impact on both the mental and temporal demand. Pharmacists expressed dissatisfaction across both mental and temporal demand, outlining the increased steps required to perform simple tasks, and an increase in administrative and staff support tasks. Nurses however, described less effort required to perform their role more effectively and efficiently.

**Table 4:** User perceptions of elements of effort expectancy extracted from nurses, doctors and pharmacists’ interviews

	Elements of effort expectancy and illustrative quote	
	Mental demand (MD)	Temporal demand (TD)
Doctors (Review & prescribe)	- Order sets have reduced the MD for simple regimens. However, there was a general lack of system flexibility, which was paradoxically often a symptom of the system attempting to improve safety (eg, by making certain tasks or viewing of screens compulsory or sequential). *• Doctor 3: ‘I think it’s quite straightforward, it’s difficult as soon as you need to alter something … because the reality is more patients are coming back for treatment and it’s when you’re having to alter cycle 2 or 3 or 5 and you have to drop this dose, change that, delay the treatment, change treatment. Which is reality for majority of patients at some stage’*. - The EMMS has introduced new steps that increases the pressure on doctor’s memory. *• Doctor 1: ‘It still requires a clinician to remember certain things and check certain things that perhaps goes against of what an intuitive path would be’.*	- The EMMS has allowed for tracking of previous prescribing and remote access has reduced TD for prescribing simple regimens. *• Doctor 4: ‘The time pressure for prescribing simple regimens and searching through paper for previous treatments has reduced, facilitated by the digital print and remote access.’* -Increased time pressure felt when troubleshooting, exacerbated by the inability to individually solve the problem. *• Doctor 2: ‘I find it very difficult to troubleshoot if I’m asked to change something by nursing or pharmacy, I often don’t know. I find it difficult to understand what I’m being asked to change’*
Pharmacists (Review & dispense)	- The increased steps to perform simple tasks are mentally draining. *• ‘Pharmacist 5: The number of steps to get one label out is just so much more. It’s just very labor intensive’.*	Greater time pressure due to: - More administrative tasks due to changed workflow *• Pharmacist 3: ‘If they don’t want something you have to reverse the repeat, re-attach it, go find the file again and like yeah, the amount of time you spend looking for stuff is ridiculous in an EMMS world’.* - Reliance of doctors to troubleshoot their prescribing issues, confirmed by a doctor’s view: *• Doctor 6: ‘I think they (pharmacists) frequently seem to prioritize those queries from us, just not sure how much of a burden it puts on them’.*
Nurses (Review & administer)	- Layout of charted medication as well as ease of access to all parameters required in treatment such as BSA and pathology has reduced the mental demand required to determine the order of administration of medication. *• Nurse 2: ‘I think it’s great in the sense that it’s all there, I can look at the bloods, I can look at the BSA I can look at the medication, it’s listed out like following a recipe’.*	- Layout and remote access allow you to better prepare for patients and reduces time pressure to complete tasks. *• Nurse 2: ‘We couldn’t prepare earlier before. We didn’t have the file in hand and sometimes you don’t have time when the file arrives. But now say the patient was not there yet, I look at their Medication Administration Record and I can communicate well, and I know what exactly I need to do’.*

### Organizational factors

#### Social influence

Amongst the nurses and pharmacists, there was a hierarchical influence of senior users on the juniors. Across all nurses there was the perception that all staff supported the EMMS (nurse 3, Table 5). On the other hand, junior pharmacists were feeling the negative influences of their seniors, highlighted by pharmacist 1, Table 5. Senior doctors were positively influencing their juniors (doctor 3, Table 5) despite some seniors, such as doctor 1 indicating ‘I’d prefer another system that I’ve used before’. Highlighting that the senior doctors were not allowing their individual perceptions to influence their junior doctors.

**Table 5:** User perceptions under the theme’s- organisational factors (social influence and facilitating condition) and individual factors (behavioural intention and Expectations based on experience related to performance and effort expectancy) from nurses, doctors and pharmacists’ interviews

Construct	User group	Illustrative quote
**Social influence**	Doctor 3	‘Our head of department has been very enthusiastic with us embracing the change there was a lot of support behind getting over the initial challenges to keep going ahead with the project’.
	Pharmacist 1	‘It can be an anxious or negative environment sometimes if my boss is moaning about it (the system)’.
	Nurse 3	‘Majority seem to like it, at the beginning it was a bit like scary, but I think we’ve all just adapted to do it and support it’.
**Facilitating condition**		
• Training	Doctor 5	‘It should be intuitive enough but it’s not. And to actually pick them up when you only do the clinic once every 2 or 3 months it’s going to be hard’. ‘I do worry that I have done something, like I’ve forgotten 3 out of the 30 steps’.
	Pharmacist 3	‘I had 3 days to learn everything and solve all problems and that wasn’t enough time’.
• Hardware	Nurse 5	‘Ergonomically it’s not very well set up. The screens are heavy and always falling forward, and everybody is going to have a bad back and a bad neck’.
**Expectations based on experience related to performance and effort expectancy**	Doctor 4	*'Remembering that cycle 1 dose reductions don’t carry over to cycle 2 and you have to do it manually…I find it dangerous’.*
	Doctor 5	*‘I don’t understand why things like vitamin b12 every 3 cycles isn’t just integrated into the pemetrexate regimen and why we have to remember, like if it’s a computer system it should be able to do those things automatically’. *
	Pharmacist 2	*‘So the system shouldn't be so prone to errors like that. If you’ve selected one, other systems I’ve used will populate for the rest of the cycles, assuming things haven’t changed, for this system to keep allowing each cycle to be different isn’t right’. *
	Nurse 5	*‘I don’t understand why things like vitamin b12 every 3 cycles isn’t just integrated into the pemetrexate regimen and why we have to remember, like if it’s a computer system it should be able to do those things automatically’.* *‘But I think the patients think the computers are safer … there’s been a few patients before we went onto the EMMS who said, well when I went to another hospital, every nurse has got a computer’.*
**Behavioral intention**	Doctor 2	‘I’m actually coming to think that the perfect EMMS might be one that doesn’t integrate well with the rest of the EMR… I think every other system has its flaws’.
	Pharmacist 5	‘If it was improved then yes so that it made life easier and safer for us.’
	Nurse 4	‘I would because I think it’s safer for the patient and not dependent on us having a piece of paper that would get lost.’

#### Facilitating condition

##### Training

Nurses expressed appreciation for the initial training and ongoing ‘elbow support’ that was provided by the health informatics team during EMMS roll-out. Doctors and pharmacists expressed varying levels of confidence and competence in using the EMMS. Difficulties were associated with the inability to comfortably use the system when dealing with complex regimens, such as being unable to ‘amend or interpret dose adjustments’. Reasons for this are described by doctor 5, Table 5. Similarly, concerns were raised by pharmacists that they weren’t provided with an adequate level of training to give them the confidence to use the EMMS (pharmacist 3, Table 5).

##### Hardware

Doctors and Pharmacists were generally satisfied with the hardware being used to support the EMMS. On the other hand, all nurses expressed concern for the ergonomics of the mobile trolleys that the computers were being supported on, as described by nurse 5 (Table 5).

### Individual factors

#### Expectations based on experience for performance and effort expectancy

This study showed that previous experience with alternate EMMS platforms at different hospitals, influenced user’s pre-defined expectations for this EMMS. Previous experience was found to influence expectations of the system related to performance and effort expectancy. Previous experience meant that users expected a degree of automation and safeguards not achieved by the current EMMS, as described by pharmacist 2 (Table 5). Expectations were associated with concerns raised by both pharmacists and doctors related to the increased reliance on a clinician memory rather than automation. There were expectations that a system would prevent ‘duplicated prescribed medications’, and include reminders such as ‘standardized vitamin b12 every 3 cycles’ as well as ‘cycle 1 dose reductions carrying over to cycle 2’, rather than putting greater reliance on prescriber’s memory as outlined by the doctors 4 and 5 (Table 5): On the other hand, nurses felt that a patient’s previous experience at other hospitals, positively influenced their perception, as described by nurse 5 (Table 5).

#### Behavioural intention

Overall, all user groups were enthusiastic about continuing to use the system in the short-term. For nurses this was attributed to the benefits they’re experiencing across all elements of the UTAUT model, illustrated by nurse 4 (Table 5). For doctors and pharmacists, the major draw point was that the system was integrated with the remainder of the hospital’s EMMS, illustrated by doctor 2 (Table 5). However, in the long-term doctors and pharmacists believed it to be only sustainable if the EMMS was ‘optimised’, illustrated by pharmacist 1 (Table 5), based on their expectations around automation and safety of the EMMS.

## Discussion

This study determined the usability of a hospital-wide EMMS implemented in a specialized Oncology unit. As multidisciplinary teams are involved in cancer-care, the three key user-groups and their inter-connected yet unique requirements were identified [23]. It illustrated that doctors and pharmacists were satisfied with the EMMS if it provided desirable utility to their practice, and nurses when the EMMS was easy to use in the nursing processes [33]. Three key themes emerged throughout the study; EMMS factors, organizational factors and individual factors. The UTAUT framework facilitated the evaluation of interrelated aspects and provided a structured summary of usability user experience factors. It allowed for cross comparisons of user groups and illustrated the key role that ‘previous experience’ plays in influencing these constructs.

This study identified the need for multi-disciplinary usability studies as the EMMS impacts individual user groups in unique ways. Outcomes of user’s views illustrated that tasks related to effort expectancy varied between the user groups. Like previous studies it was identified that aspects of the EMMS layout, configuration and output quality can reduce the mental energy required of searching for important information and the time taken to achieve this [34]. This is highlighted in the contrast in acceptance between nurses with doctors and pharmacists’. It illustrated that doctors and pharmacists EMMS dissatisfaction was related to the system not providing desirable utility to their specific practice, unlike nurses who identified benefits to their work practices [33]. Similarly, our study highlighted that managerial satisfaction was due to the benefits of automated auditing [7], thus reducing the effort expectancy, which has previously been found to be a key driver for uptake and support [21, 34].

The UTAUT framework facilitated a systematic comparison between user-groups, allowing for evaluation of interrelated aspects. The UTAUT highlights the importance of contextual analysis in developing strategies for technology implementation within organisations [27]. Despite the ability of the existing models to predict intention and usage, current theoretical perspectives on individual acceptance are notably weak demonstrating the need for qualitative investigations [27]. The interrelated nature of facilitating condition and its impact on user groups demonstrated this. A lack of understanding of system functionality often results in independent troubleshooting by users, skipping steps or entering information in the EMMS differently to overcome system barriers [2]. By doctors not initially appreciating the source of the problem when prescribing, in a strongly multidisciplinary unit, it had downstream consequences for other user’s (pharmacists and nurses) workflow. The findings are consistent with those of previous studies that illustrate the role of facilitating conditions in user acceptance [35, 36]. Considerations should be made when system redesign isn’t possible, to incorporate lessons learned from the troubleshooting incidents into the training of staff to improve usability across all groups [37].

User’s previous experience with other EMMS resulted in pre-defined expectations of the EMMS, [27]. The difference in expectations between user groups who had had previous experience with an Oncology EMMS on a different platform at other hospitals (doctors and pharmacists) and those who didn’t (nurses), showed a difference in user acceptance. Palm et al. also demonstrated that confirmation of expectations was strongly associated with doctors’ and nurses’ satisfaction [33]. Previous studies have found that the main required system expectations were the possibility of searching data, security and confidentiality protocols, the availability of data analytic tools and collecting data about different types of cancer [23]. Achieving these expectations can potentially be achieved by the application of user centred design which has been shown to be more efficient and more usable than when not initially considered in the design [38]. However, user-centred design can also be limited by the nature of an integrated system. Whilst integrated systems facilitate the availability of information in one place, they also limit the customisability thus impeding innovation and potentially impacting on the usability in specialized settings [39, 40]. Interestingly, the UTAUT theorises ‘experience’ as a moderating influencer on facilitating condition, social influence and effort expectancy [27]. Our findings illustrated that experiences with a ‘best-of-breed’ system influenced performance and effort expectations of the integrated system.

The finding that performance expectancy can impact on the intention to use the new EMMS aligns with previous studies that claim performance expectancy is an important determinant of doctors’ intention to use a new technology [32]. The greatest benefit and also dis-benefit on performance expectancy for doctors was introduced by pre-defined order sets. Our findings are consistent with other studies that found a key facilitator for user-acceptance were order sentences that increased prescriber efficiency [21, 34] and evidence-based practices [6, 21, 41]. However, we also identified oncology specific issues related to protocol-mandated care. A major limitation in flexibility was found when doctors attempted to order outside the order sets [2] and when adjusting doses or frequencies. The perception of the system was that it lacked the flexibility required in the oncology setting, which was paradoxically often a symptom of systems attempting to improve safety [34]. Like previous studies [6], automaticity contributed to a negative user experience to a degree [2], as users (doctors and pharmacists) felt reduced efficacy was being facilitated by a greater number of mouse clicks [21] and effort were required to prescribe or review medications outside of standardised order sets.

Limitations of this study are that it was conducted in a single-centre site and was conducted 6 months following EMMS implementation, therefore initial user resistance issues that may have arisen during the shakedown phase had not been overcome or ironed out. It is currently unknown if these issues would remain after more time using the system. Evidence suggests that it may take up to 2 years post-implementation until the unit returns to complete stability as EMMS optimization can be an iterative process [42], and up to 4 years before there is a return on investment [34]. Furthermore, the full EMMS had not been implemented at the time of the study, with only 70% of the protocols integrated into the EMMS creating a hybridized environment. We also did not directly measure safety or efficiency quantitatively, and perceptions around these domains can differ substantially from objective assessments.

## Conclusion

Our research has identified that different user groups had different usability needs from an EMMS implemented in the Oncology setting. Nurses demonstrated overall satisfaction with the EMMS, whilst doctors and pharmacists indicated that changes were required to better meet their needs. The greatest usability problems were related to restricted automaticity and system complexity, which hindered user uptake and EMMS success. Doctors and pharmacists indicated short-term system acceptance however questioned the long-term sustainability if usability issues weren’t addressed. The study demonstrates the feasibility and utility of the UTAUT framework to evaluate usability of an EMMS for multiple user groups in the Oncology setting.

## Supplementary Information

**Additional file 1.** Interview guide.

## Data Availability

The datasets used and/or analysed during the current study are available from the corresponding author on reasonable request.

## Abbreviations

EMMS: Electronic Medication Management System
UTAUT: Unified Theory and Use of Technology
PE: Performance expectancy
EE: Effort Expectancy
SI: Social influence
FC: Facilitating conditions
BI: Behavioural intention
MAR: Medication Administration Record
