# Supplementary material for: Usability evaluation of an integrated electronic medication management system implemented in an oncology setting using the unified theory of the acceptance and use of technology
Source: BMC Med Inform Decis Mak. 2021 Jan 6;21:4. doi: 10.1186/s12911-020-01348-y (PMC7789263; doi:10.1186/s12911-020-01348-y)
Supplement: Supplementary file 1 — Additional file 1. Interview guide. [file 12911_2020_1348_MOESM1_ESM.docx]

**Additional File 1:**

Interview guide

1. **Effort Expectancy:**
   1. What is it about the EMMS that you find easy to use?
   2. In general, what is it about the EMMS that you find difficult to use?
   3. Describe what your interaction is like using the EMMS?
   4. Describe difficulties you faced in becoming skilful in using the EMMS?
2. **Performance Expectancy:**
   1. In what ways do you see using the EMMS useful in your job?
   2. Can you explain your thoughts on the quality of information provided by the EMMS?
   3. In what ways do you see using the EMMS impacts your productivity?
   4. Describe the impact the EMMS has on the time it talks to accomplish your tasks.
3. **Facilitating Condition:**
   1. What support has been made available to you since go live? Comment of each type of support.
   2. Describe the support resources that have been made available to you, do you find you have the resources necessary to use the system?
   3. How confident are you in your own ability to adapt to the new way of working with EMMS?
   4. To what extent is the new way of working with the EMMS compatible with other systems you use.
4. **Social Influence:**
   1. Generally speaking, who are the colleagues that are important and influential to your work?
   2. What do they think about the new way of working using the EMMS?
   3. In what way do their opinions change your opinions about the new way of working using EMMS?
5. **Behavioural intention:**
   1. If you had a choice, would you continue to use the EMMS? Why/why not?
